# Polymorphisms Influence the Expression of the Fas and FasL Genes in COVID-19

**DOI:** 10.3390/ijms26020666

**Published:** 2025-01-14

**Authors:** Wandrey Roberto dos Santos Brito, William Botelho de Brito, Fabiane dos Santos Ferreira, Emmanuelle Giuliana Mendes Santana, Jeferson da Costa Lopes, Ednelza da Silva Graça Amoras, Sandra Souza Lima, Erika Ferreira dos Santos, Flávia Póvoa da Costa, Kevin Matheus Lima de Sarges, Marcos Henrique Damasceno Cantanhede, Mioni Thieli Figueiredo Magalhães de Brito, Andréa Luciana Soares da Silva, Mauro de Meira Leite, Maria de Nazaré do Socorro de Almeida Viana, Fabíola Brasil Barbosa Rodrigues, Rosilene da Silva, Giselle Maria Rachid Viana, Tânia do Socorro Souza Chaves, Adriana de Oliveira Lameira Veríssimo, Mayara da Silva Carvalho, Daniele Freitas Henriques, Carla Pinheiro da Silva, Juliana Abreu Lima Nunes, Iran Barros Costa, Igor Brasil-Costa, Juarez Antônio Simões Quaresma, Izaura Maria Vieira Cayres-Vallinoto, Leonardo Oliveira Reis, Luiz Fábio Magno Falcão, Eduardo José Melo dos Santos, Antonio Carlos Rosário Vallinoto, Maria Alice Freitas Queiroz

**Affiliations:** 1 Laboratory of Virology, Institute of Biological Sciences, Federal University of Pará, Belém 66075-110, Brazil; wandrey.ben1@gmail.com (W.R.d.S.B.); william-brito@hotmail.com (W.B.d.B.); fabianedsferreira@gmail.com (F.d.S.F.); emmanuelle.santana@icb.ufpa.br (E.G.M.S.); jefersonlopesbio@gmail.com (J.d.C.L.); ednelza@hotmail.com (E.d.S.G.A.); sandra.souza.lima@gmail.com (S.S.L.); ivallinoto@ufpa.br (I.M.V.C.-V.); vallinoto@ufpa.br (A.C.R.V.); 2 Graduate Program in Biology of Infectious and Parasitic Agents, Institute of Biological Sciences, Federal University of Pará, Belém 66075-110, Brazil; erikaferreira.bio@gmail.com (E.F.d.S.); flaviacosta@ufpa.br (F.P.d.C.); kevsarges@gmail.com (K.M.L.d.S.); ma_cantanhede@hotmail.com (M.H.D.C.); maryvyana@hotmail.com (M.d.N.d.S.d.A.V.); fbrasil.barbosa@yahoo.com.br (F.B.B.R.); rosilenesilva2005@yahoo.com.br (R.d.S.); ejmsantos@yahoo.com (E.J.M.d.S.); 3 Laboratory of Genetics of Complex Diseases, Institute of Biological Sciences, Federal University of Pará, Belém 66075-110, Brazil; mionibrito@gmail.com (M.T.F.M.d.B.); dealuciana@gmail.com (A.L.S.d.S.); mauroleite@gmail.com (M.d.M.L.); 4 Laboratory of Basic Research on Malaria, Parasitology Section, Evandro Chagas Institute, Health and Environment Surveillance Secretariat, Brazilian Ministry of Health, Ananindeua 66093-020, Brazil; giselleviana@iec.gov.br (G.M.R.V.); tania.chaves@uol.com.br (T.d.S.S.C.); 5 School of Medicine, Institute of Medical Sciences, Federal University of Pará, Belém 66075-110, Brazil; 6 Belém Adventist Hospital, Belém 66093-904, Brazil; adylameira@gmail.com (A.d.O.L.V.); myhcarvalho07@gmail.com (M.d.S.C.); 7 Arbovirology and Hemorrhagic Fevers Section, Evandro Chagas Institute, Health and Environment Surveillance Secretariat, Brazilian Ministry of Health, Ananindeua 66093-020, Brazil; danielehenriques@iec.gov.br (D.F.H.); carlapinheiro@iec.gov.br (C.P.d.S.); 8 Laboratory of Immunology, Section of Virology, Instituto Evandro Chagas, Health and Environment Surveillance Secretariat, Brazilian Ministry of Health, Ananindeua 66093-020, Brazil; julianalima@iec.gov.br (J.A.L.N.); irancosta@iec.gov.br (I.B.C.); igorcosta@iec.gov.br (I.B.-C.); 9 Graduate Program in Virology, Evandro Chagas Institute, Department of Science, Technology, Innovation and Strategic Health Inputs, Ministry of Health of Brazil, Ananindeua 66093-020, Brazil; juarez.quaresma@gmail.com; 10 Center of Biological and Health Sciences, University of the State of Pará, Belém 66087-670, Brazil; fabiofalcao@uepa.br; 11 UroScience, Faculty of Medical Sciences, State University of Campinas, Campinas 13083-590, Brazil; reisleo@unicamp.br; 12 ImmunOncology, Pontifical Catholic University of Campinas, Campinas 13060-904, Brazil

**Keywords:** COVID-19, long COVID, FAS, FASL, polymorphisms, gene expression, cytokines

## Abstract

The apoptotic molecule Fas and its ligand FasL are involved in the process of T-lymphocyte death, which may lead to lymphopenia, a characteristic of severe coronavirus disease 2019 (COVID-19). In this study, we investigated the influence of polymorphisms in the *FAS* and *FASL* genes, *FAS* and *FASL* gene expression, and plasma cytokine levels on COVID-19 severity and long COVID occurrence. A total of 116 individuals with severe COVID-19 and 254 with the non-severe form of the disease were evaluated. In the post-COVID-19 period, samples from 196 individuals with long COVID and 67 from people who did not have long COVID were included. Genotyping and quantification of gene expression were performed via real-time PCR, and cytokine measurement was performed via flow cytometry. The AA genotype for *FAS* rs1800682 (A/G) and the TT genotype for *FASL* rs763110 (C/T) were associated with increased *FAS* and *FASL* gene expression, respectively (*p* < 0.005). Higher plasma IFN-γ levels were associated with higher *FAS* and *FASL* gene expression (*p* < 0.05). Among individuals with non-severe COVID-19, carriers of the AA genotype for *FAS* rs1800682 (A/G) had higher levels of *FAS* expression, more symptoms, and higher IFN-γ levels (*p* < 0.05). No association of the evaluated markers with long COVID were observed. The AA genotype of *FAS* rs1800682 (A/G) and the TT genotype of *FASL* rs763110 (C/T) influence the levels of *FAS* and *FASL* gene expression. Higher gene expression of *FAS* and *FASL* may lead to greater inflammation in COVID-19 patients, with higher levels of IFN-γ and T lymphocyte death.

## 1. Introduction

Severe acute respiratory syndrome coronavirus 2 (SARS-CoV-2) is responsible for the coronavirus disease 2019 (COVID-19) pandemic, which has led to the death of millions of people worldwide. In addition, COVID-19 can lead to the development of severe debilitating sequelae, even after resolution of the infection [1,2,3]. SARS-CoV-2 infection can trigger mild to moderate symptoms, or it can have severe manifestations [4]. Severe COVID-19 is associated with several factors, such as sex, advanced age, the presence of comorbidities, and an exacerbated immune response [5,6].

Patients who have recovered from COVID-19 may still have symptoms for months after the end of the infection, resulting in a syndrome called long COVID [7,8]. Symptoms such as fatigue/weakness, shortness of breath, mental confusion, dizziness, headache, muscle pain, anosmia, ageusia, chronic cough, and chest pain have been identified in patients with this condition [7,9,10,11]. Both individuals who had severe COVID-19 and those who had mild manifestations may develop long COVID, which is characterized by the persistence of an inflammatory response [10,12].

The adaptive and innate immune systems are stimulated during SARS-CoV-2 infection [13]. In COVID-19, the immune response of lymphocytes is mediated mainly by CD4^+^ T cells, which differentiate into type 1 T helper (Th1) cells and are responsible for producing antiviral cytokines such as interferon-gamma (IFN-γ) [14,15,16]. A more accelerated response of specific CD4^+^ T cells has been related to mild cases of COVID-19, while the absence or prolonged reduction of these cells has been identified in severe or fatal cases [15,17,18].

T-cell lymphopenia is a mechanism associated with the severity of COVID-19, which may occur owing to the apoptosis of these cells [19,20,21,22]. A positive correlation between Fas (CD95/Apo-1) molecule expression and the plasma level of soluble Fas ligand (FasL) in patients with COVID-19 has already been associated with T-cell lymphopenia [20].

Fas is a cell membrane receptor that, after binding with its ligand, FasL, stimulates apoptotic signaling in target cells [23,24,25,26]. This occurs because Fas-FasL binding induces the signaling of caspase-8, a master regulator of cellular apoptosis pathways, which causes nuclear DNA cleavage and the proteolysis of vital cellular proteins [26,27].

Activation of the Fas-FasL pathway contributes to determining the evolution of several infections. Dysregulated activation of the Fas-FasL pathway may lead to excessive neuroinflammation during HSV-1 infection [28], liver damage in chronic HCV infection [29], and dengue-virus-induced vascular endothelial cell destruction [30], as well as contribute to the development of tuberculosis [31]. In SARS-CoV-2 infection, André et al. (2022) demonstrated that T-cell apoptosis is responsible for T lymphopenia in individuals with severe COVID-19 [20]. An experimental study of SARS-CoV-2 infection in mice showed that inhibition of FasL increased animal survival and reduced cell death and inflammation. Furthermore, critically ill COVID-19 patients had higher levels of FasL in bronchoalveolar lavage [32]. This shows the importance of activation of apoptosis mediated by the Fas-FasL pathway in the pathogenesis of COVID-19.

Several studies have investigated the association of *FAS* and *FASL* polymorphisms with infectious agents, since genetic variations can alter the expression of *FAS* and *FASL* genes and promote inappropriate activation of apoptosis [33,34,35,36,37,38,39,40,41,42]. The *FAS* rs1800682 (A/G) and *FASL* rs5030772 (A/G) polymorphisms have been associated with higher proviral load and the development of human T-cell lymphotropic virus type 1 (HTLV-1)-associated [36,37,38] and with the progression of human immunodeficiency virus type 1 (HIV-1) infection and disease development [39,40]. The *FASL* rs763110 (C/T) polymorphism has been shown to influence hepatitis B virus (HBV) infection through alteration in hepatocyte apoptosis [41], and the *FAS* rs2234767 (G/A) polymorphism has shown a protective effect against the occurrence of hepatocellular carcinoma in HBV infection [42]. In neoplasias, the polymorphic alleles for *FAS* rs1800682 (A/G) and *FASL* rs763110 (C/T) represented a protective effect against gastric cancer [43]; the *FA*S rs1800682 (A/G), *FASL* rs5030772 (A/G), and *FASL* rs763110 (C/T) polymorphisms were associated with the risk of breast cancer [44,45], and the wild-type genotype for *FASL* rs763110 (C/T) was considered a risk factor for prostate pathologies [46].

However, little is known about the occurrence of polymorphisms in the *FAS* and *FASL* genes and their influence on the inflammatory process in SARS-CoV-2 infection. Thus, this study investigated the association of *FAS* rs1800682 (A/G), *FAS* rs223476 (G/A), *FASL* rs763110 (C/T), and *FASL* rs5030772 (A/G) polymorphisms with the severity of COVID-19 and the development of long COVID, with the aim of identifying whether genetic factors in apoptosis molecules could contribute to the differences in clinical manifestations observed among people infected with SARS-CoV-2, enabling a better understanding of the pathogenesis of the disease. These polymorphisms were selected because they have already been associated with other diseases.

## 2. Results

### 2.1. Frequency of Polymorphisms in the FAS and FASL Genes in Individuals with COVID-19 and Long COVID

Among individuals with COVID-19, those with severe manifestations had a mean age of 51.5 years (standard deviation [SD]: 13.6), and the majority were male (58.7%). The individuals with non-severe COVID-19 had a mean age of 43.8 years (SD: 13.1), and the majority were female (58.48%). Among individuals with COVID-19, 137 (37%) had comorbidities (diabetes mellitus, cardiovascular disease, and obesity).

Analysis of the polymorphisms revealed that there was no association of the variants *FAS* rs1800682 (A/G) and *FAS* rs2234767 (G/A) with the severity of COVID-19. The TT genotype (*p* = 0.0144; *OR* = 2.45; 95% CI: 1.24–4.84) and T allele (*p* = 0.0061; *OR* = 1.61; 95% CI: 1.15–2.24) of the *FASL* rs763110 (C/T) polymorphism were associated with severe COVID-19; however, analysis of genotypes and alleles for the *FASL* rs5030772 (A/G) polymorphism revealed no significant differences between the severe and non-severe COVID-19 groups (Table 1).

In the post-COVID-19 period, individuals with long COVID had a mean age of 45.4 years (SD: 12.1), and the majority were female (59.3%). Individuals without long COVID had a mean age of 38.9 years (SD: 9.9), and females represented 51.2% of the sample. The evaluation of the frequencies of *FAS* and *FASL* gene polymorphisms showed no significant differences between the group with long COVID and the group without long COVID (Table 2). The frequency of polymorphisms was evaluated according to the presence and absence of comorbidities; however, none of the analyses showed a significant association (Table S1).

These initial results showed that in the context of COVID-19, the FASL rs763110 (C/T) polymorphism could represent a risk for disease severity, but not for long COVID, possibly because long COVID is manifested by clinical manifestations of different types and intensities.

### 2.2. Gene Expression Levels of FAS and FASL in Individuals with COVID-19 and Long COVID

**Figure 1:**
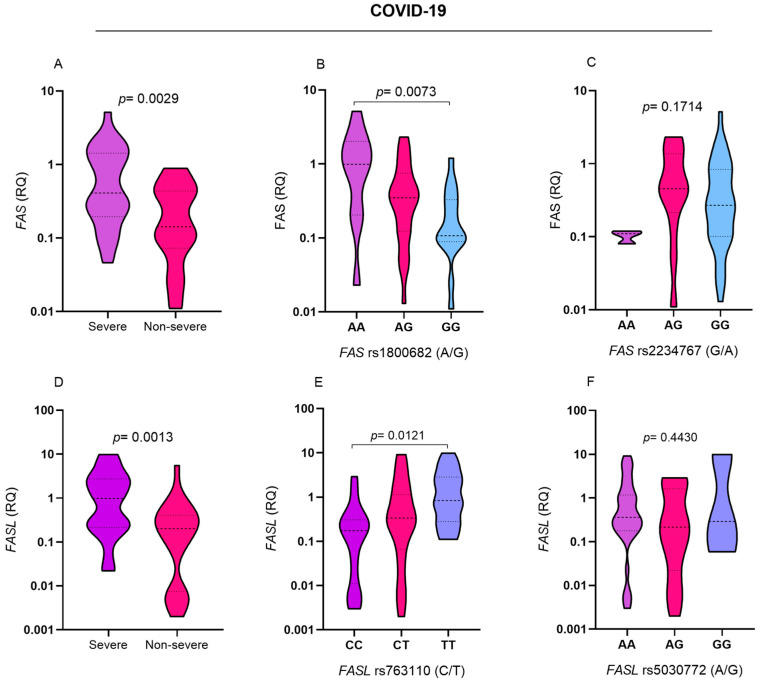
Evaluation of *FAS* and *FASL* gene expression levels in patients with COVID-19. Comparison of *FAS* expression levels between (**A**) individuals with severe and non-severe COVID-19 and individuals carrying different genotypes for the (**B**) *FAS* rs1800682 (A/G) and (**C**) *FAS* rs2234767 (G/A) polymorphisms. Comparison of *FASL* expression levels between (**D**) individuals with severe and non-severe COVID-19; individuals carrying different genotypes for the (**E**) *FASL* rs763110 (C/T) and (**F**) *FASL* rs5030772 (A/G) polymorphisms.

*FAS* gene expression levels were greater in individuals with severe COVID-19 than in those with non-severe symptoms (*p* = 0.0029; Figure 1A). In the evaluation of *FAS* expression in relation to polymorphisms, expression levels were greater in individuals with the AA genotype for the *FAS* rs1800682 (A/G) polymorphism than in individuals with the GG genotype (*p* = 0.0073; Figure 1B). There was no significant difference in *FAS* expression levels among individuals with different genotypes for the *FAS* rs2234767 (G/A) polymorphism (Figure 1C). The *FASL* gene expression levels were greater in the group of individuals with severe COVID-19 than in those with non-severe symptoms (*p* = 0.0013; Figure 1D). The *FASL* gene expression levels were greater in TT genotype carriers than in CC genotype carriers for the *FASL* rs763110 (C/T) polymorphism (*p* = 0.0121; Figure 1E). There was no significant difference in *FASL* expression levels among individuals carrying the different genotypes for the *FASL* rs5030772 (A/G) polymorphism (Figure 1F). *FAS* and *FASL* gene expression levels were assessed according to the presence and absence of comorbidities (Figure S1) and among individuals with different types of comorbidities (diabetes mellitus, cardiovascular disease, and obesity) and individuals without these conditions (Figure S2); however, none of the analyses showed a significant association. These results suggest that, when assessed through bivariate analyses, the genetic variations *FAS* rs1800682 (A/G) and *FASL* rs763110 (C/T) may influence the increased expression of *FAS* and *FASL*, respectively, and contribute to the severity of COVID-19.

**Figure 2:**
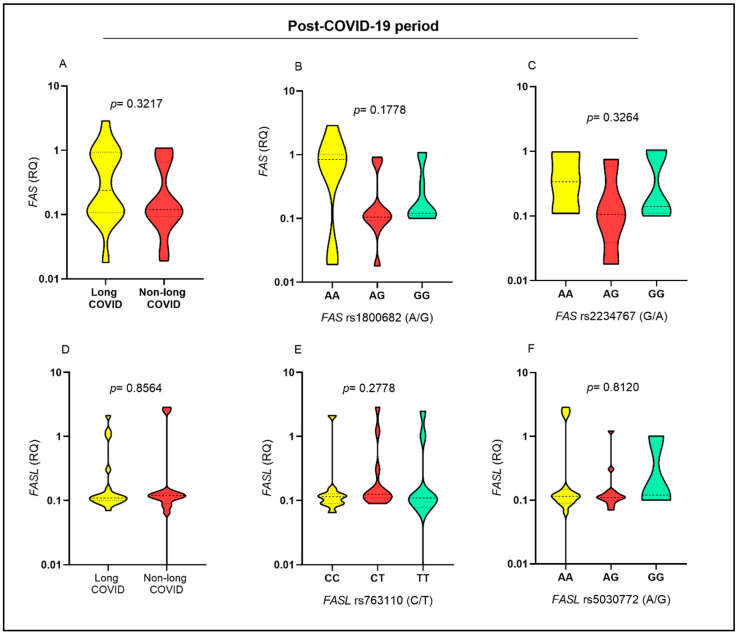
Evaluation of *FAS* and *FASL* gene expression levels in the post-COVID-19 period. Comparison of FAS expression levels between (**A**) individuals with and without long COVID and individuals carrying different genotypes for the (**B**) *FAS* rs1800682 (A/G) and (**C**) *FAS* rs2234767 (G/A) polymorphisms. Comparison of *FASL* expression levels between (**D**) individuals with and without long COVID; carrying different genotypes for the (**E**) *FASL* rs763110 (C/T) and (**F**) *FASL* rs5030772 (A/G) polymorphisms.

The *FAS* gene expression levels were not significantly different between individuals with and without long COVID (Figure 2A) or between individuals carrying different genotypes for the *FAS* rs1800682 (A/G) (Figure 2B) and *FAS* rs2234767 (G/A) (Figure 2C) polymorphisms. *FASL* expression levels were not associated with long COVID (Figure 2D) or with the *FASL* rs763110 (C/T) (Figure 2E) and *FASL* rs5030772 (A/G) (Figure 2F) polymorphisms. The lack of association between *FAS* and *FASL* levels and long COVID, as well as the expression levels of these molecules with the genetic polymorphisms evaluated, suggest that the immune response triggered during long COVID may not have the activation of the *FAS* and *FASL* pathway as its main mechanism.

### 2.3. Plasma Cytokine Levels in Individuals with COVID-19 and Long COVID

**Figure 3:**
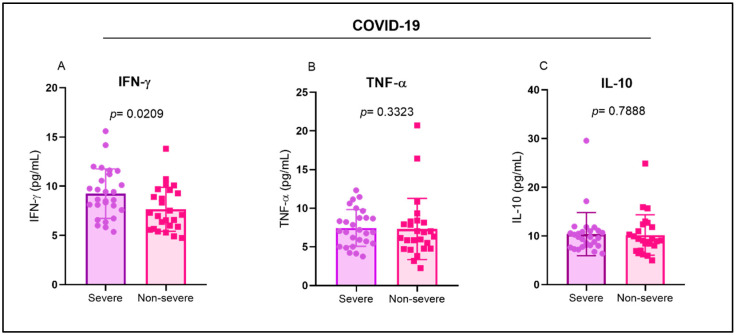
Evaluation of cytokine levels in patients with COVID-19. Comparison of the levels of the cytokines (**A**) IFN-γ, (**B**) TNF-α, and (**C**) IL-10 between groups of individuals with severe and non-severe manifestations of the disease.

IFN-γ levels were higher in the group with severe COVID-19 than in the group with non-severe symptoms (Figure 3A). In contrast, the levels of TNF-α and IL-10 were not significantly different between the severe and non-severe COVID-19 groups (Figure 3B,C).

**Figure 4:**
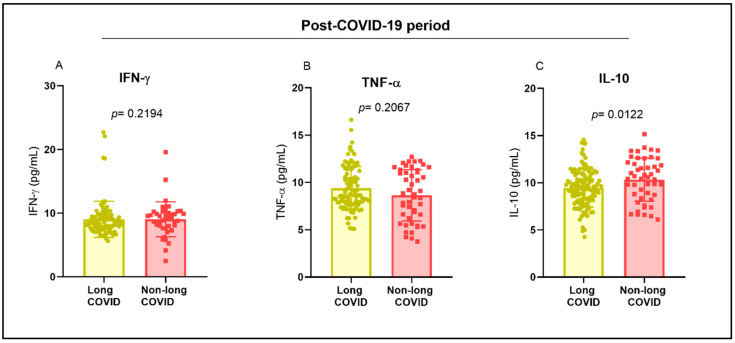
Evaluation of cytokine levels in the post-COVID-19 period. Comparison of the levels of the cytokines (**A**) IFN-γ, (**B**) TNF-α, and (**C**) IL-10 between groups of individuals with and without long COVID.

In the post-COVID-19 period, the levels of IFN-γ, TNF-α, and IL-10 were not significantly different between the groups with and without long COVID (Figure 4A,B). IL-10 levels were higher in the group of individuals without long COVID (Figure 4C).

### 2.4. Correlation Between FAS, FASL, and IFN-γ Expression Levels in Individuals with COVID-19

**Figure 5:**
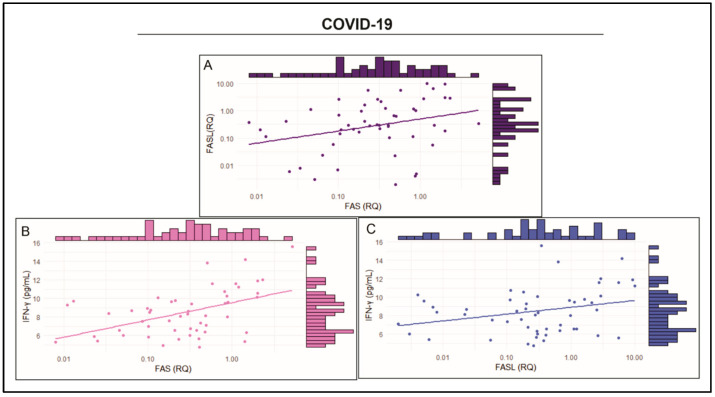
Correlations between (**A**) FAS and FASL expression levels (*r* = 0.392; *p* = 0.0257); (**B**) FAS expression and IFN-γ plasma levels (*r* = 0.4603; *p* = 0.0006); and (**C**) FASL expression and IFN-γ plasma levels (*r* = 0.2624; *p* = 0.060).

Analysis of the correlation of *FAS* and *FASL* expression and IFN-γ plasma levels in individuals with COVID-19 revealed slight positive correlations between *FAS* and *FASL* expression levels (*r* = 0.392; *p* = 0.0257; Figure 5A), between *FAS* expression and IFN-γ plasma levels (*r* = 0.4603; *p* = 0.0006; Figure 5B), and between *FASL* expression and IFN-γ plasma levels (*r* = 0.2624; *p* = 0.0602; Figure 5C).

### 2.5. Multiple Evaluation of Variables in Individuals with COVID-19

Multiple logistic regression was used to assess whether the investigated variables were associated with the severity of COVID-19. However, the results showed that the variables were not associated with the severity of COVID-19, showing that in the presence of other immunological components, none of the polymorphisms influence the clinical evolution of the disease (Table 3).

Multiple linear regression analysis revealed an association of the increased FAS gene expression levels with wild-type AA genotype for the *FAS* rs1800682 (A/G) polymorphism (β: 0.924; 95% CI: 0.423–1.425; *p* = 0.0005). An increase in *FAS* expression levels was associated with increased plasma levels of IFN-γ (β: 0.162; 95% CI: 0.083–0.232; *p* = 0.0001). The increased FASL expression levels were associated with the TT polymorphic genotype for *FASL* rs763110 (C/T) (β: 1.539; 95% CI: 0.175–2.903; *p* = 0.0278). *FASL* expression levels were associated with increased plasma IFN-γ levels (β: 0.488; 95% CI: 0.2436–0.717; *p* = 0.0001). *FASL* expression levels decreased with increasing IL-10 plasma levels (β: −0.164; 95% CI: −0.308–−0.0210; *p* = 0.0250). These results show that regardless of the clinical manifestation of COVID-19, the expression levels of *FAS* and *FASL* appear to be influenced by the genetic variations *FAS* rs1800682 (A/G) and *FASL* rs763110 (C/T), respectively, even in the presence of other immunological components (Table 4).

**Figure 6:**
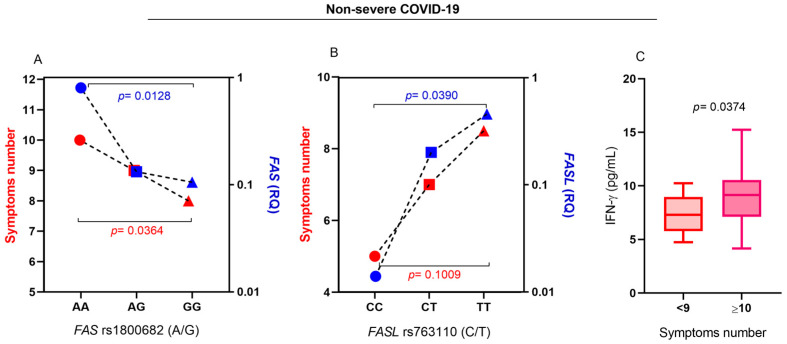
Comparison of the (**A**) median number of symptoms (in red) and the levels of FAS gene expression (in blue) among carriers of the genotypes for the FAS rs1800682 (A/G) polymorphism; (**B**) median number of symptoms (in red) and the levels of FASL gene expression (in blue) among carriers of the genotypes for the FASL rs763110 (C/T) polymorphism; (**C**) IFN-γ plasma levels according to the number of symptoms. Symptoms: fever, cough, runny nose, pain behind the eyes, headache, sore throat, chest pain, abdominal pain, body aches, nausea, vomiting, diarrhea, shortness of breath, weakness, tiredness, anosmia, and weight loss.

As the variables evaluated in the present study were not associated with the severity of COVID-19 in the multiple logistic regression, and based on the results of the multiple linear regression analysis that showed that the AA genotype for the *FAS* rs1800682 (A/G) polymorphism and TT genotype for *FASL* rs763110 (C/T) had higher levels of *FAS* and *FASL* gene expression, respectively, we evaluated this relationship with the number of clinical symptoms of COVID-19 in the group of individuals with the non-severe form of the disease, which showed that carriers of the AA (Figure 6A) and TT (Figure 6B) genotypes had a greater number of symptoms compared to carriers of other genotypes. Individuals with more symptoms had higher IFN-γ plasma levels (Figure 6C). These results show that, although the *FAS* rs1800682 (A/G) and *FASL* rs763110 (C/T) variations have not been identified as risk factors for the development of severe COVID-19, they may contribute to greater morbidity among non-severe individuals and may have a significant impact on the lives of these people. The description of the statistical variables in Figure 6 is shown in Tables S2–S4. The frequency of symptoms in individuals with non-severe COVID-19 is described in Table S5.

## 3. Discussion

In viral infections, FAS–FASL interaction can eliminate infected cells by activating apoptotic processes and contribute to the assembly of the antiviral response through the production of cytokines and chemokines, which act in the inflammatory response and in the maintenance of immunological tolerance [30,47,48]. In this context, this study evaluated whether polymorphisms in the FAS and FASL genes may be associated with the severity of COVID-19 and the development of long-term COVID-19.

The frequencies of the *FAS* rs1800682 (A/G), *FAS* rs2234767 (G/A), and *FASL* rs5030772 (A/G) polymorphisms were not associated with the severity of COVID-19, unlike the findings of studies on other viral infections, where the high frequency of the FAS rs1800682 (A/G) polymorphism was associated with a high proviral load in individuals with HTLV-1 who had HTLV-1-associated myelopathy (HAM), and the FAS rs1800682 (A/G) polymorphism was considered a possible risk factor for progression to HAM [36,37]. The *FAS* rs1800682 (A/G) polymorphism was also shown to be associated with the clinical manifestations of adult T-cell leukemia/lymphoma (ATLL) [49]. In HIV-1 infection, the *FAS* rs1800682 (A/G) and *FAS* rs2234767 (G/A) polymorphisms were associated with the apoptosis of CD4+ T lymphocytes [39] and disease progression in people living with HIV-1 [40]. However, none of these three polymorphisms were associated with HBV or HCV infection [50,51]. Thus, the frequencies of the *FAS* rs1800682 (A/G), *FAS* rs2234767 (G/A), and *FASL* rs5030772 (A/G) polymorphisms seem to have different patterns for each type of viral infection. These differences may be related to specific characteristics of the pathogenesis of each of these infections or may be influenced by the sample size evaluated.

According to the bivariate analysis, a high frequency of the *FASL* rs763110 polymorphism (C/T) was associated with the severity of COVID-19. Other studies that performed this type of analysis showed different relationships of the *FASL* rs763110 (C/T) polymorphism with other infections; the highest frequency of the polymorphism was associated with HCV infection [51,52] and disease progression in women with HPV infection [53]. The mutation in FASL rs763110 (C/T) may mediate the development of the disease in HCV infection due to structural changes in the mRNA because of its interference in mRNA transcription [52]. In contrast, the *FASL* rs763110 (C/T) polymorphism conferred protection against cirrhosis in individuals with HBV [50]. The analysis of the frequency of the *FASL* rs763110 (C/T) polymorphism did not show an association with herpes simplex virus type 2 (HSV-2) infection in women [54] or with the development of HAM in people living with HTLV-1 [37]. However, in the multiple logistic regression analysis, in which the polymorphism was evaluated together with other investigated factors, the frequency of the *FASL* rs763110 (C/T) polymorphism was no longer significant for the severity of COVID-19, indicating that other factors are more important in the progression of SARS-CoV-2 infection, and in this context, the polymorphism is no longer relevant.

The quantification of gene expression revealed that individuals with severe COVID-19 had higher levels of *FAS* and *FASL* expression. Fas and FasL have already been identified as crucial factors for the progression of COVID-19 and lethality [32,55]. Higher *FAS* expression on CD4^+^ and CD8^+^ T cells was identified in patients with COVID-19 than in healthy controls [20,56]. Thus, the increased gene expression of *FAS* and *FAS*L may be a determining factor for the severity of COVID-19, as it is likely that they trigger the death of T cells and thus lead to the development of lymphopenia present in patients with severe COVID-19.

The binding of FasL to its Fas receptor on the T-cell membrane triggers a caspase activation pathway, with binding to caspase-8 and consequent cleavage of caspase-3, which results in cell apoptosis [26,57]. Individuals with severe COVID-19 had higher FASL expression and higher caspase-8 activity in CD4^+^ and CD8^+^ T cells [20,35]. Thus, the higher expression of *FAS* and *FASL* on the surface of T lymphocytes, induced by SARS-CoV-2 infection, may contribute to triggering apoptosis of these cells, leading to the development of lymphopenia. Individuals with COVID-19 who present lymphopenia associated with lung alterations are more likely to die [58].

The AA genotype of the *FAS* rs1800682 (A/G) and the TT genotype of the *FASL* rs763110 (C/T) were associated with higher *FAS* and *FASL* expression, respectively, in the bivariate analyses and in the multiple linear regression analysis. These results support the evidence of the influence of these genotypes on the expression of *FAS* and *FASL* molecules and, possibly, on increased activation of apoptosis, which contributes to more severe COVID-19 symptoms. In other viral infections, such as that caused by HCV, the TT genotype of *FASL* rs763110 (C/T) has already been associated with a risk of infection progression; however, there has been no investigation of *FASL* gene expression [51].

The evaluation of plasma levels of TNF-α, IFN-γ, and IL-10 showed that although IFN-γ levels were higher in individuals with the severe form of COVID-19 in the bivariate analysis, this result was not confirmed in the multiple logistic regression analysis. Nevertheless, higher levels of IFN-γ were associated with the expression of *FAS* and *FASL* according to multiple linear regression and correlation analyses. IFN-γ is a proinflammatory cytokine with broad immunomodulatory capacity that can activate local immune cells, interrupting the viral replication process in several steps, in addition to other antiviral functions [59,60,61,62]. Studies have shown that IFN-γ regulates the expression of Fas and FasL molecules on the surface of T cells during infection by other pathogens, thus playing a role in apoptotic processes [63,64,65]. On the other hand, in vitro studies have already shown that IFN-γ may be responsible for the development of pulmonary immunopathology in patients with SARS [66]. Thus, the positive correlation between the expression levels of *FAS* and *FASL* and these molecules with the levels of FN-γ suggests that an accentuated production of these components may promote the inflammatory process in the lungs and lead to the development of more intense symptoms of COVID-19.

Elevated levels of IFN-γ have been observed in hospitalized patients with COVID-19 compared with healthy individuals [67], as well as in individuals with severe COVID-19 relative to mild cases in Iran [68], but no differences were detected between patients hospitalized in the intensive care unit (ICU) and those not hospitalized in the ICU [67], nor among individuals with severe and mild manifestations of COVID-19 in China [69], similar to our study. These results suggest that the IFN-γ response is influenced by other factors in COVID-19 that influence cytokine production and need to be further investigated. This variation in IFN-γ levels between populations contributes to the cytokine not being a good marker for determining COVID-19 severity.

In our study, there was no significant difference in plasma levels of TNF-α and IL-10 between the groups with severe and non-severe COVID-19. However, elevated plasma levels of TNF-α have been identified in individuals with severe COVID-19 in previous studies [66,69,70], and higher levels of IL-10 have been observed in patients with COVID-19 compared to healthy individuals and also in severe cases of the disease compared to non-severe cases [22,67,71,72]. It is likely that this difference in results may be related to the time of infection and also to the altered production of certain cytokines. Early production of IL-10 in SARS-CoV-2 infection has been associated with the severity of COVID-19 [72], since elevated levels of IL-10 can influence the production of cytokines responsible for the resolution of the infection. However, it was not possible to determine the time of infection of the individuals evaluated in our study.

Despite the fact that multivariate analysis did not show a relationship between the investigated genetic polymorphisms, *FAS* and *FASL* expression levels, and cytokine levels with the severity of COVID-19, the strong association of high levels of *FAS* gene expression with the AA genotype for the *FAS* rs1800682 (A/G) polymorphism and of high levels of *FASL* expression with the TT genotype for *FASL* rs763110 (C/T) and of these with the high levels of IFN-γ observed in COVID-19 prompted us to evaluate whether these factors could influence the number of clinical symptoms of the disease among those with the non-severe form of COVID-19. The results showed that, in addition to carriers of the AA and TT genotypes presenting higher levels of *FAS* and *FASL*, respectively, these individuals presented a greater number of symptoms, and the greater number of symptoms was associated with higher levels of IFN-γ. It is possible that the AA genotype of *FAS* rs1800682 (A/G) and TT for *FASL* rs763110 (C/T) may contribute to inducing a greater expression of *FAS* and *FASL* and, together with IFN-γ, cause a more intense inflammatory process, resulting in a greater number of symptoms in individuals with the non-severe form of COVID-19. As described in other studies, the AA genotype for *FAS* rs1800682 (A/G) and TT for *FASL* rs763110 (C/T) may lead to greater symptomatology/progression of diseases caused by different viral agents [38,53].

This is one of the first studies to evaluate the frequency of *FAS* and *FASL* polymorphisms with the severity of COVID-19. In this study, polymorphisms in the *FAS* and *FASL* genes were not directly associated with the severity of COVID-19, but it was possible to identify that the *FAS* rs1800682 (A/G) and *FASL* rs763110 (C/T) variations can influence the expression levels of FAS and FASL and contribute to the number of COVID-19 symptoms, showing that these polymorphisms can be considered one of the factors that influence, in part, the pathogenesis of SARS-CoV-2 infection, although it is not a determinant of the severity of the disease. However, our study evaluated polymorphisms only in a mixed population from the Brazilian Amazon (with contributions from whites, blacks, and indigenous people) [73] and did not evaluate other ethnic groups. Therefore, additional studies in other population strata with different genetic characteristics and the evaluation of variations in other genes in the apoptosis pathway could complement the understanding of the relevance of polymorphisms in the *FAS* and *FASL* genes in the development of COVID-19 manifestations and their potential use as a biomarker of disease progression.

The frequencies of the *FAS* rs1800682 (A/G), *FAS* rs2234767 (G/A), *FASL* rs5030772 (A/G), and *FASL* rs763110 (C/T) polymorphisms were not associated with long COVID. Furthermore, the polymorphisms were not associated with variations in *FAS* and *FASL* expression levels. Long COVID is characterized by various dysfunctions of the body and has been associated with a chronic inflammatory response in certain tissues, which is mediated mainly by the activation of the cGAS–STING pathway and IFN-I production [70]. Thus, genetic variations in the *FAS* and *FASL* genes do not seem to contribute to changes in the immune response in long COVID. However, the lack of association of polymorphisms in long COVID needs to be further investigated through longitudinal studies, which would allow us to assess whether polymorphisms could influence the evolution of long COVID symptoms over time.

This study provides an initial understanding of the role of genetic variations in the *FAS* and *FASL* genes in SARS-CoV-2 infection. However, this study only evaluated two polymorphisms in the *FAS* gene and two polymorphisms in the *FASL* gene, but there are other polymorphisms in these genes that need to be evaluated in COVID-19. In addition, this study did not evaluate other components of the apoptosis cascade, since several components are involved in this process and variations in other genes may influence the intensity of apoptosis activation. The lack of this information represents a limitation of this study.

This study provides an initial understanding of the role of genetic variations in the FAS and FASL genes in SARS-CoV-2 infection. However, this study has some limitations, including the evaluation of only two polymorphisms in the *FAS* gene and two polymorphisms in the *FASL* gene, but there are other polymorphisms in these genes that need to be evaluated in COVID-19; this study did not evaluate other components of the apoptosis cascade, since multiple components are involved in this process, and variations in other genes may influence the intensity of apoptosis activation. In addition, it was not possible to evaluate the direct relationship between the lymphocyte count of the patients and the polymorphisms investigated.

## 4. Materials and Methods

### 4.1. Study Population and Sample Collection

The present study included blood samples from 370 individuals with COVID-19, 116 with severe COVID-19, and 254 with non-severe clinical manifestations (mild and moderate COVID-19). The clinical classification was performed according to the criteria established by the World Health Organization [4].

A total of 263 individuals were evaluated during the post-COVID-19 period; 196 had a clinical diagnosis of long COVID (dyspnea, chest pain, muscle weakness, tremor, fatigue, myalgia, headache, visual changes, and insomnia), and 67 did not have long COVID. Long COVID diagnosis was based on the presence of symptoms that persisted for at least three months after resolution of the infection and that had no specific cause and/or relationship with other morbidities. People admitted to the group without long COVID were followed up for 6 months after the resolution of the acute infection to confirm the absence of symptoms related to the syndrome.

This study included individuals of both sexes, aged 18 years or older, who were not vaccinated against SARS-CoV-2 and were treated at the COVID-19 outpatient clinic of the University of the State of Pará, Belém Adventist Hospital or Evandro Chagas Institute, from July 2020 to May 2021. The group of long COVID patients included those who sought care at the long COVID outpatient clinic of the University of the State of Pará. Individuals with COVID-19 who reported co-infections, cancer, or autoimmune disease were excluded from the study.

Blood samples (10 mL) were collected by venipuncture with a vacuum collection tube containing ethylenediaminetetraacetic acid (EDTA) as an anticoagulant. The samples were transported to the Laboratory of Virology, Federal University of Pará, where they were processed for separation of plasma and leukocytes. Leukocyte samples were used for DNA extraction, and plasma samples were used for the determination of plasma cytokines.

### 4.2. DNA Extraction

The extraction of DNA from leukocytes from whole-blood samples was performed with a Puregene™ kit (Gentra Systems, Inc., Minneapolis, MN, USA) following the manufacturer’s protocol, which consisted of the steps of cell lysis, protein precipitation, and precipitation and hydration of the DNA. After extraction, the obtained DNA was quantified via spectrophotometric reading on a BioDrop™ instrument (Bio-Rad, Hercules, CA, USA) following the protocol recommended by the manufacturer [31].

### 4.3. Genotyping of FAS rs1800682 (A/G), FAS rs2234767 (G/A), FASL rs763110 (C/T), and FASL rs5030772 (A/G)

The genotypes of the investigated polymorphisms were identified via real-time PCR with the StepOnePLUS™ Real-Time PCR System (Thermo Fisher, Carlsbad, CA, USA). The reactions consisted of the use of the following commercially available TaqMan™ assays: FAS rs1800682 (C___9578811_10), FAS rs2234767 (C__12123966_10), FASL rs763110 (C___3175437_10), and FASL rs5030772 (C__32334221_10) (Thermo Fisher, Carlsbad, CA, USA). These assays contained primers and probes specific for the amplification of the target sequence. The reaction mixture consisted of 1X MasterMix, H_2_O, 20X assay buffer and 50 ng of DNA, which was subjected to the following cycling conditions: 10 min at 95 °C and 40 cycles of 15 s at 95 °C and 1 min at 60 °C [31].

### 4.4. RNA Extraction and Reverse Transcription

Total RNA was extracted from peripheral blood leukocytes via the TRIzol™ Plus RNA Purification Kit total RNA extraction kit (Thermo Fisher Scientific, Waltham, MA, USA). The steps followed the protocol recommended by the manufacturer. The concentration of the extracted RNA was determined with the use of a BioDrop™ (Bio-Rad, Hercules, CA, USA) according to the manufacturer’s instructions. All the total RNA samples for the synthesis of complementary DNA (cDNA) had concentrations equal to 50 ng/µL [31].

The synthesis of cDNA from RNA was performed via the “High-Capacity cDNA Reverse Transcription^®^ with RNase Inhibitor” kit (Applied Biosystems, Foster City, CA, USA). For each reaction, a mixture with a final volume of 20 µL was prepared, containing 2 µL of 10X RT Buffer, 0.8 µL of 25X dNTP mixture (100 nM), 2 µL of random primer, 1 µL of MultiScribeTM Reverse Transcriptase, 1 µL of RNaseOUTTM, and 3.2 µL of ultrapure water supplied by the kit and then 10.0 µL of extracted RNA. The mixture was subsequently placed in a Mastercycler Personal thermal cycler (Eppendorf, Hamburg, Germany) and subjected to cycles of 25 °C for 10 min, 37 °C for 120 min, and 85 °C for 5 min [31].

### 4.5. Quantification of Gene Expression

Gene expression was quantified via real-time PCR. Initially, the quantitative (qPCR) reactions with the cDNAs and probes (endogenous and target genes) were standardized to calculate the efficiency of the amplification reactions. In the standardization reactions, different concentrations of cDNA were tested (pure and in 4 dilutions of factor 2: 1:2, 1:4, 1:8, and 1:16). All reactions were performed on plates in triplicate, and the same cDNA (at different dilutions) was analyzed with the different probes to construct an efficiency curve to validate the 2^−ΔΔCT^ analysis method. All tests showed efficiency as expected (100% ± 10) [74].

The relative quantification of gene expression consisted of amplification of the target gene with the endogenous gene (normalizer) using TaqMan™ assays (Applied Biosystems, Foster City, CA, USA) and the StepOnePLUS™ Real-Time PCR System (Thermo Fisher Scientific, Waltham, MA, USA). The reactions were performed in singleplex format according to the manufacturer’s protocol. The assay used was Hs00236330_m1 for *FAS* and Hs00181226_g1 for *FASL*; glyceraldehyde-3-phosphate dehydrogenase (GAPDH) was used as an endogenous control (Hs02786624_g1). All assays were obtained commercially (Thermo Fisher Scientific, Waltham, MA, USA). For the reaction, 5 µL of 2X TaqMan^®^ Universal PCR Master Mix, 0.5 µL of 20x TaqMan Gene Expression Assay, 1 µL of cDNA, and 10.5 µL of RNAse-free water were used, with the following thermocycling conditions: 2 min at 50 °C, followed by 10 min at 95 °C and 1 min at 60 °C.

The relative quantification (RQ) of the expression of target genes was determined using the comparative CT method (ΔΔCt) and the formula 2^−ΔΔCT^, where ΔΔCt = ΔCt sample − ΔCt reference (Life Technologies, Carlsbad, CA, USA) [31].

### 4.6. Plasma Measurement of Cytokine Levels

The plasma levels of the cytokines IFN-γ, TNF-α, and IL-10 were measured by flow cytometry with the Human Th1/Th2/Th17 Cytometric Bead Array (CBA) Kit (BD Biosciences, San Diego, CA, USA) and BD FACS Canto II equipment. The manufacturer’s guidelines were followed for all procedures. The methodology used is based on beads conjugated with capture antibodies, in which six populations of beads, with different fluorescence intensities, conjugated to a capture antibody specific for each cytokine, were mixed to form the CBA and then read in the −3 FL channel on the flow cytometer.

### 4.7. Statistical Analysis

The genotypic and allelic frequencies of the polymorphisms were estimated by direct counting, and the significance of the differences between the studied groups was calculated by the χ2 (chi-squared) test, Fisher’s exact test, and G test. The Hardy–Weinberg test was performed to evaluate whether the observed genotypic frequency distributions were in accordance with expectations. The Shapiro–Wilk test was used to analyze the normality of the distribution of *FAS*, *FASL*, and cytokine expression levels. The comparison of *FAS*, *FASL*, and cytokine expression levels among the investigated groups was performed using the nonparametric Mann–Whitney, Kruskal–Wallis, and Spearman correlation tests. Multiple logistic regression was used to evaluate the associations of the investigated variables with the severity of COVID-19. Multiple linear regression analysis was performed to evaluate the associations between FAS, FASL, and IFN-γ gene expression and the variables investigated. Differences with a *p* value ≤ 0.05 were considered significant. All tests were performed with BioEstat software5.3 and GraphPad Prism 5.0.

### 4.8. Ethical Aspects

The project was approved by the National Research Ethics Committee (CAEE: 33470020.1001.0018), protocol number 2.190.330. The individuals were informed about the objectives of the study, and those who agreed to participate in the study signed an informed consent form and responded to a clinical-epidemiological questionnaire.

## 5. Conclusions

In summary, the present study showed that polymorphisms in the FAS and FASL genes are not associated with the severity of COVID-19, but the AA genotype of FAS rs1800682 (A/G) and the TT genotype of FASL rs763110 (C/T) influenced the increased expression levels of FAS and FASL, respectively, and the increased expression levels of *FAS* and *FASL* associated with elevated levels of IFN-γ may contribute to intensifying the inflammatory process and the development of COVID-19 symptoms, since the AA genotype for FAS rs1800682 (A/G) and the TT genotype for FASL rs763110 (C/T) were associated with a greater number of disease symptoms among patients with non-severe COVID-19. Although these preliminary results are relevant for understanding the mechanisms involved in the development of COVID-19 symptoms, future investigations are needed to evaluate the gaps considered limiting in our study, in addition to other polymorphisms in genes involved in the inflammatory response, as this information may contribute to the identification of biomarkers that can identify individuals with a greater propensity to develop the severe form of COVID-19.

## Figures and Tables

**Table 1:** Comparison of genotypic and allelic frequencies of the investigated polymorphisms in the *FAS* and *FASL* gene between the severe COVID-19 and non-severe COVID-19 groups.

Genotypes and Alleles	Severe COVID-19 (n = 116) n (%)	Non-Severe COVID-19 (n = 254) n (%)	*p*	*OR* (95% CI)
***FAS* rs1800682 (A/G)** **(−670)**				
AA	22 (19.0)	49 (19.3)	0.9949 ^a^	
AG	56 (48.3)	123 (48.4)		
GC	38 (32.8)	82 (32.3)		
* A	0.43	0.44	0.9824 ^a^	
* G	0.57	0.56		
***FAS* rs2234767 (G/A)** **(−1377G)**				
AA	1 (0.9)	7 (2.8)	1.0000 ^b^	
AG	29 (25)	49 (19.3)		
GG	86 (74.1)	198 (78.0)		
* A	0.13	0.12	0.8064 ^a^	
* G	0.87	0.88		
***FASL* rs763110 (C/T)** **(−844)**				
CC	50 (43.1)	141 (55.5)	0.0253 ^a^	
CT	46 (39.7)	90 (35.4)		
TT	20 (17.2)	23 (9.1)		
TT vs. CC			0.0144 ^a^	2.45 (1.24–4.84)
* C	0.74	0.60	0.0061 ^a^	1.61 (1.15–2.24)
* T	0.26	0.40		
***FASL* rs5030772 (A/G)** **(IVS2nt −124)**				
AA	93 (80.2)	211 (83.1)	0.1991 ^b^	
AG	19 (16.4)	40 (15.7)		
GG	5 (4.3)	3 (1.2)		
* A	0.88	0.91	0.2039 ^a^	
* G	0.12	0.09		

n = number of individuals; * allele; ^a^ chi-squared test; ^b^ G test. *OR*, *odds ratio*; CI, confidence interval.

**Table 2:** Comparison of genotypic and allelic frequencies of the investigated polymorphisms in the *FAS* and *FASL* gene between the long COVID and no long COVID groups.

Genotypes and Alleles	Long COVID (n = 196) n (%)	No long COVID (n = 67) n (%)	*p*
***FAS* rs1800682 (A/G)** **(−670)**			
AA	38 (19.4)	16 (23.9)	0.6947 ^a^
AG	91 (46.4)	28 (41.8)	
GG	67 (34.2)	23 (34.3)	
* A	0.43	0.45	0.7356 ^b^
* G	0.57	0.55	
***FAS* rs2234767 (G/A)** (−1377)			
AA	4 (2.0)	1 (1.5)	0.9446 ^c^
AG	37 (18.9)	12 (17.9)	
GG	155 (79.1)	54 (80.6)	
* A	0.11	0.10	0.8664 ^b^
* G	0.89	0.90	
***FASL* rs763110 (C/T)** (−844)			
CC	94 (48.0)	37 (55.2)	0.5668 ^a^
CT	79 (40.3)	24 (35.8)	
TT	23 (11.7)	6 (9.0)	
* C	0.68	0.73	0.3269 ^b^
* T	0.32	0.27	
***FASL* rs5030772 (A/G)** (IVS2nt −124)			
AA	165 (84.2)	55 (82.1)	0.9510 ^c^
AG	29 (14.8)	11 (16.4)	
GG	3 (1.5)	1 (1.5)	
* A	0.92	0.90	0.9119 ^b^
* G	0.08	0.10	

n = number of individuals; * allele; ^a^ chi-squared test; ^b^ Fisher’s exact test; ^c^ G test.

**Table 3:** Multiple logistic regression analysis of the variables investigated in relation to the severity of COVID-19.

Variables	*OR*	95% CI	*p*
**Severity**			
*FAS* rs1800682 (A/G)			
AA	1.424	0.073–2.769	0.3507
AG	3.845	0.051–2.862	0.9938
GG	Ref		
*FASL* rs763110 (C/T)			
CC	Ref		
TC	2.642	0.024–2.886	0.5097
TT	5.980	0.003–9.124	0.2753
*FAS* expression (RQ)	1.047	0.744–1.473	0.0816
*FASL* Expression (RQ)	2.223	0.953–5.184	0.0642
IFN-γ (pg/mL)	1.024	0.598–1.755	0.9296
TNF-α (pg/mL)	1.359	0.867–2.132	0.1807
IL-10 (pg/mL)	8.252	0.556–1.224	0.3401

*OR*: *odds ratio*; CI: confidence interval.

**Table 4:** Multiple linear regression analysis of *FAS* and *FASL* gene expression levels in patients with COVID-19.

Variables	Estimate	95% CI	*p*
***FAS* gene expression (RQ)**			
*FAS* rs1800682 (A/G)			
AA	0.924	0.423–1.425	0.0005
AG	0.094	−2.470–1.004	0.6266
GG	Ref		
*FAS* rs2234767 (G/A)			
AA	Ref		
AG	−0.207	−1.754–1.338	0.7876
GG	−0.904	−2.444–0.634	0.2424
IFN-γ (pg/mL)	0.162	0.083–0.232	0.0001
TNF-α (pg/mL)	−0.029	−0.094–0.036	0.3711
IL-10 (pg/mL)	0.066	0.021–0.110	0.4464
***FASL* gene expression (RQ)**			
*FASL* rs763110 (C/T)			
CC	Ref		
CT	0.9061	−0.372–2.184	0.1604
TT	1.539	0.175–2.903	0.0278
*FASL* rs5030772 (A/G)			
AA	−1.331	−3.685–1.022	0.2606
AG	−2.317	−4.761–0.127	0.0626
GG	Ref		
IFN-γ (pg/mL)	0.480	0.243–0.717	0.0001
TNF-α (pg/mL)	−0.053	−0.219–0.111	0.5156
IL-10 (pg/mL)	−0.164	−0.308–0.021	0.0250

CI: confidence interval.

## Data Availability

The data are available from the corresponding author upon reasonable request.

## Supplementary Materials

The following supporting information can be downloaded at: https://www.mdpi.com/article/10.3390/ijms26020666/s1.
