# Supplementary material for: Polymorphisms Influence the Expression of the Fas and FasL Genes in COVID-19
Source: Int J Mol Sci. 2025 Jan 14;26(2):666. doi: 10.3390/ijms26020666 (PMC11765610; doi:10.3390/ijms26020666)
Supplement: Supplementary file 1 [file ijms-26-00666-s001.zip › ijms-3345863-supplementary.pdf]

Table S1. Assessment of the frequency of polymorphisms according to the presence and absence of comorbidities.

**Genotypic Profile**	**No Comorbidities (n= 233) n (%)**	**Comorbidities (n= 137) n (%)**	*p*
***FAS* rs1800682 (A/G) (-670)**			
AA	37 (15.9)	33 (24.1)	
AG	109 (46.8)	63 (46.0)	0.1079
GC	87 (37.3)	41 (29.9)	
***FAS* rs2234767 (G/A)** (-1377)			
AA	6 (2.6)	2 (1.5)	
AG	56 (24.0)	24 (17.5)	0.2441
GG	171 (73.4)	111 (81.0)	
***FASL* rs763110 (C/T)** (-844)			
CC	102 (43.8)	73 (53.3)	
CT	96 (41.2)	46 (33.6)	0.2050
TT	35 (15.0)	18 (13.1)	
***FASL* rs5030772 (A/G) (IVS2nt -124)**			
AA	191 (82.0)	116 (84.7)	
AG	39 (16.7)	18 (13.1)	0.5553
GG	3 (1.3)	3 (2.2)	

Table S2. Description of statistical variables in Figure 6A.

Genotypic Profile *FAS* rs1800682 (A/G)	Number of Symptoms Median (IQR)	*FAS* Expression Levels Median (IQR)
AA	10 (7)	0.8025 (0.1570)
AG	9 (6)	0.132 (0.3078)
GG	8 (6)	0.1055 (0.1710)

IQR: interquartile range.

Table S3. Description of statistical variables in Figure 6B.

Genotypic Profile *FASL* rs763110 (C/T)	Number of Symptoms Median (IQR)	*FASL* Expression Levels Median (IQR)
CC	5 (4)	0.0140 (0.0600)
CT	7 (6)	0.2010 (0.2815)
TT	8.5 (8.5)	0.4585 (0.4848)

IQR: interquartile range.

Table S4. Description of statistical variables in Figure 6C.

Number of Symptoms	IFN-γ Levels Median (IQR)
<9	6.57 (3.18)
>10	8.91 (3.11)

IQR: interquartile range.

Table S5. Frequency of symptoms present in individuals with non-severe COVID-19 (n=254).

Symptoms	n	%
Fever	187	73.6
Cough	169	66.5
Runny nose	110	43.3
Pain behind the eyes	70	27.6
Headache	172	67.7
Sore throat	137	53.9
Chest pain	126	49.6
Abdominal pain	69	27.2
Body ache	175	68.9
Nausea	84	33.1
Vomiting	42	16.5
Diarrhea	114	44.9
Shortness of breath	135	53.1
Weakness	120	47.2
Tiredness	160	63.0
Anosmia	155	61.0
Weight loss	158	62.2

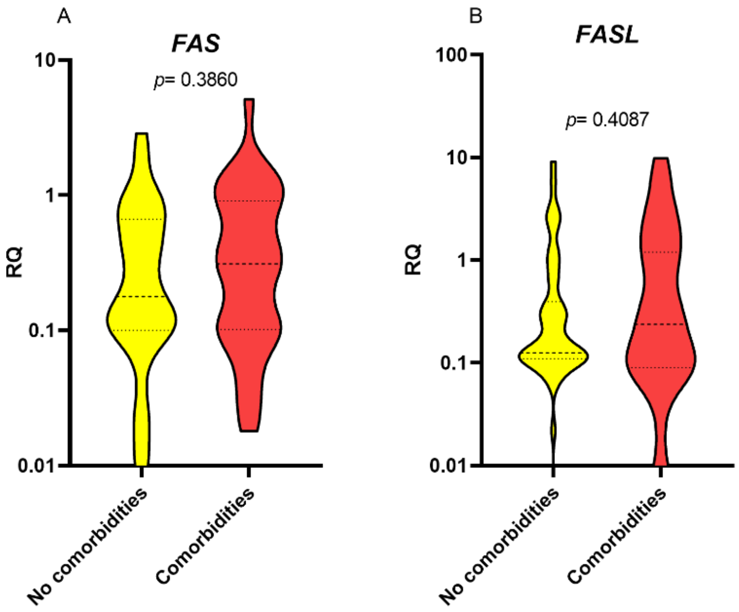

Figure S1. Assessment of FAS and FASL gene expression levels according to the presence and absence of comorbidities.

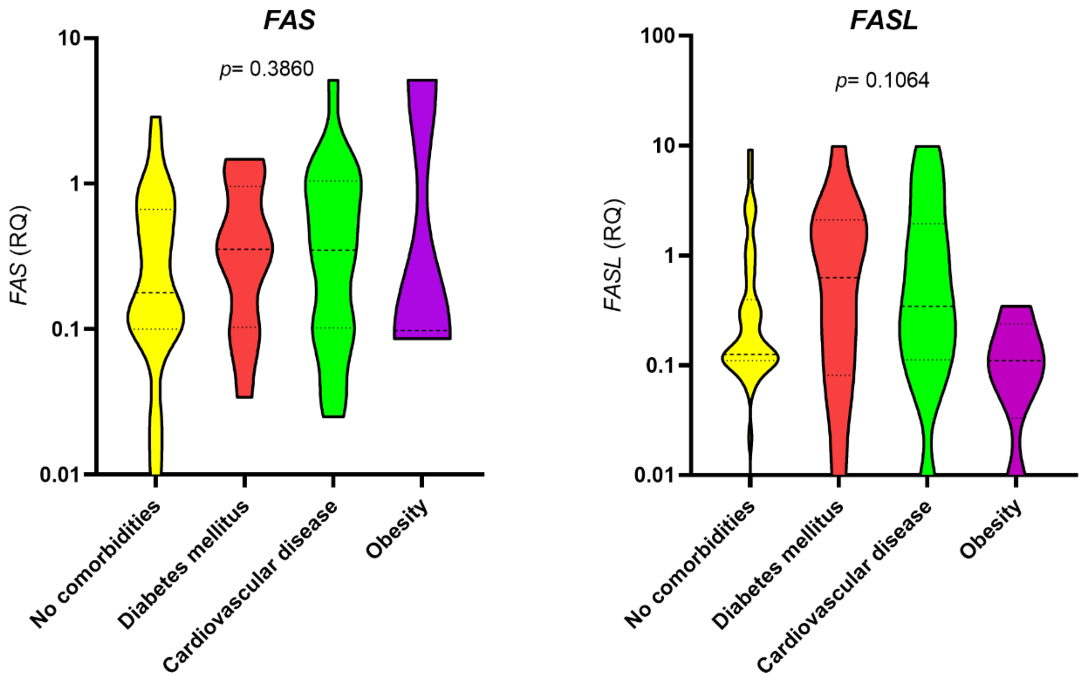

Figure S2. Evaluation of FAS and FASL gene expression levels among individuals with different types of comorbidities and individuals without these conditions.
